# Predictive Utility of the Multi-Process Action Control Framework for Self-Reported and Device-Measured Physical Activity Behavior of Adolescents

**DOI:** 10.3390/bs14090841

**Published:** 2024-09-19

**Authors:** Denver M. Y. Brown, Carah D. Porter, Christopher Huong, Claire I. Groves, Matthew Y. W. Kwan

**Affiliations:** 1 Department of Kinesiology, Kansas State University, 1105 Sunset Ave, Manhattan, KS 66502, USA; carahporter@ksu.edu; 2 Department of Psychology, The University of Texas at San Antonio, 1 UTSA Circle, San Antonio, TX 78249, USA; christopher.huong@my.utsa.edu (C.H.); claire.groves@my.utsa.edu (C.I.G.); 3 Department of Child and Youth Studies, Brock University, 1812 Sir Isaac Brock Way, St. Catharines, ON L2S 3A1, Canada; mkwan@brocku.ca

**Keywords:** accelerometry, youth, theory, high school, identity, exercise

## Abstract

Understanding the correlates of physical activity behavior is imperative for informing the development of interventions to address the low rates of physical activity guideline adherence among adolescents living in the United States. This cross-sectional study examined the predictive utility of the Multi-Process Action Control (M-PAC) framework for explaining self-reported and device-measured physical activity behavior among a Hispanic-majority sample of adolescents. A total of 1849 high school students (mean age = 16.0 ± 1.22 SD years; 52.3% women; 87.8% Hispanic) enrolled in one school district in south-central Texas completed a survey including instruments to assess M-PAC framework constructs (instrumental and affective attitudes, perceived capability and opportunity, behavioral regulation, habit, identity) and moderate-to-vigorous physical activity (MVPA) behavior. A subsample (*n* = 435) wore accelerometers for seven days. The results from robust linear regression models revealed role identity and habit were significant predictors of self-reported MVPA. Role identity was a significant predictor of accelerometer-derived daily MVPA and raw acceleration in the most active hour but not daily raw acceleration. The findings indicated reflexive processes are robust predictors of adolescent physical activity and should be the focus of interventions designed to promote adoption and maintenance of physical activity during this developmental life stage.

## 1. Introduction

Adolescence is generally considered a healthy period in a person’s life. However, many noncommunicable diseases that emerge later in life are partly the result of modifiable risk behaviors established during this time, such as physical inactivity [1]. The importance of physical activity for healthy development during adolescence cannot be overstated, yet it remains a significant public health challenge [2]. Physical activity is known to enhance cardiovascular health, improve muscle and bone strength, and benefit mental health by reducing symptoms of anxiety and depression [3]. Despite these well-documented benefits, a majority of adolescents worldwide fail to meet the public health recommendations of 60 min of moderate-to-vigorous physical activity (MVPA) on average each day [4]. Population-level data indicate that physical activity engagement peaks in early adolescence but declines across adolescence before stabilizing at inadequate levels in adulthood [5,6]. Such behavioral patterns are problematic in light of research that has shown that trajectories of physical activity behavior track reasonably well from adolescence into adulthood (e.g., those who become inactive during adolescence tend to stay inactive during adulthood) [7,8]. The adolescent years therefore represent a unique window of opportunity for shaping lifelong physical activity habits and thus promoting health across the lifespan. Moving forward, it is imperative that we improve our understanding of the processes underlying the adoption and maintenance of physical activity behavior during this key developmental stage for the purpose of informing intervention development to address the ongoing public health epidemic of physical inactivity and its associated consequences [9,10].

Although physical activity engagement may be best understood through a socioecological lens that considers the complex interplay between individuals, their environment and the policies that shape these environments [11], intrapersonal psychological processes at the individual level represent influential and proximal factors underlying behavior. To date, much of the literature investigating the psychological correlates of physical activity among adolescent samples have used social cognitive theories, including many couched within the theory of planned behavior [12]. Meta-analytic evidence from 23 studies demonstrated that social cognitive theories account for an average of 24–37% of the variance in adolescent physical activity behavior and have an even greater explanatory power for intention (48%; [12]). A key shortcoming of several social cognitive theories, however, is that intention formation (i.e., motivation to act) is positioned as the proximal determinant of physical activity behavior. Yet, the evidence from adult samples indicates that nearly half (47.6%; 38.7% successful intenders versus 33.0% unsuccessful intenders) of individuals with intentions to engage in physical activity are unsuccessful at translating their cognitions into action [13], otherwise known as the intention–behavior gap [14]. Some recent findings suggest that intention–behavior discordance may be lower among adolescents (i.e., 37.8% successful intenders versus 12.8% unsuccessful intenders; [15]). Importantly, motivation is a necessary condition for people to be active. That is, without the intention to be physically active, very few individuals (i.e., <5%) actually engage in physical activity behaviors [13,15].

Several extensions of social cognitive theories integrating post-intentional processes have been advanced in an attempt to explain why intentions to engage in physical activity are not always successful [16]. Initial action control theories integrated regulatory processes as key constructs that aid in reducing intention–behavior discordance [17]. Regulatory processes are behavioral, cognitive, and affective tactics that individuals undertake to translate their intentions into physical activity behavior (e.g., self-monitoring, action/coping planning, goal setting). More recently, reflexive processes (automaticity [habit] and identity) have been included in these models [18] as automatic and less conscious behaviors and cognitions resulting from repeated action control over time. The introduction of regulatory and reflexive processes has played an instrumental role in advancing our current understanding of the volitional phase of physical activity behavior. As a result, the Health Action Process Approach [17] and Multi-Process Action Control (M-PAC) framework [18] have been commonly drawn upon over the past decade to develop theory-informed interventions, aiming to promote the adoption and maintenance of physical activity behavior [19,20]. The M-PAC framework is unique in that it incorporates three hierarchically organized layers of processes that can be used to explain physical activity behavior in a progressive manner from intention formation to behavioral maintenance. At its foundation are reflective processes, which represent the consciously deliberated expectations of physical activity engagement, and collectively facilitate intention formation. These include instrumental attitudes, affective attitudes, perceived capability, and perceived opportunity. Regulatory processes make up the next layer in the hierarchy, promoting the adoption of physical activity behavior through translating intention into action. Behavioral regulation tactics have been described as the metaphorical glue between physical activity intentions and behavior [21], taking hold long enough until reflexive processes, situated at the pinnacle of the M-PAC framework (i.e., habit and identity), take over and facilitate the long-term maintenance of physical activity through repeated action control. While the integration of reflexive processes into broader models of physical activity behavior is relatively recent, it is gaining traction, evident in the emergence of dual-process models involving reflective and reflexive processes [22,23,24]. In summary, the M-PAC framework’s tripartite approach of integrating reflective, regulatory, and reflexive processes provides an ordered structure through which physical (in)activity can be understood and intervened upon for individuals with different needs along the continuum from developing strong intentions to sustaining physical activity across the lifespan.

Despite its recent development, the M-PAC framework has received considerable attention for explaining the variance in physical activity behavior among several different populations including adults [25,26], new parents [27], individuals with poor mental health [28], and those diagnosed with cancer [29], among others (for a review, see [27]). Far fewer studies have examined the M-PAC framework’s predictive utility for adolescent physical activity [15,30,31]. This is surprising given the framework’s inclusion of constructs proposed to play a critical role in sustaining physical activity over time (e.g., habit, identity), which, as evidence indicates, many adolescents struggle with as their priorities and responsibilities change with aging [6]. Furthermore, adolescence is recognized as a crucial period in which one’s sense of identity becomes coherent and thus represents a promising target for intervention [32]. Given that school environments could support the development of a strong physical activity identity through promoting multiple physical activity opportunities (e.g., athletics, recess, physical education), it is not unreasonable to hypothesize that physical activity identity may be one of the most robust correlates of physical activity behavior during this life stage. This notion is supported by the cross-sectional findings of [31] in that identity along with habit were the strongest predictors of MVPA behavior among a sample of 1176 Canadian adolescents in Grade 11. Together, the reflexive processes explained an additional 8.2% of the unique variance in adolescent physical activity behavior beyond the 5.1% and 13.2% explained by regulatory and reflective processes, respectively. Given that only one study examined the explanatory power of the M-PAC framework for adolescent physical activity to date, further work is warranted to determine whether these results replicate among different samples and with device-assessed physical activity metrics.

Recently, in the broader field of psychology, there has been a notable increase in the prevalence of replication and extension studies [33,34] aiming to validate and build upon existing research findings. Yet, within kinesiology and exercise psychology, replication efforts have been limited [35], despite concerns that replicability has been overlooked [36]. Furthermore, much of the testing of theories to explain physical activity behavior, including the M-PAC framework, has relied on self-reported physical activity instruments. These instruments are known to have limitations in terms of reliability and validity [37]; therefore, it is imperative to determine whether findings can be replicated with device-based measures, which can improve the precision and accuracy of physical activity estimates [38]. Issues surrounding measurement error may be further compounded when using the M-PAC framework to explain self-reported physical activity given its inclusion of identity as a behavioral antecedent. Specifically, reporting a strong exercise identity has been found to be associated with greater inflated self-reported estimates of exercise frequency when both measures are included within the same survey [39]. While device-based measures are not without their shortcomings [40,41], reducing measurement error can help with gaining a better understanding of which constructs are the strongest correlates of actual physical activity behavior, which can help refine theories of physical activity behavior and better inform intervention development.

Beyond replication, it is also important that we aim to build upon our current knowledge through extending existing studies. Identity was observed to be one of the strongest predictors of physical activity behavior among adolescents within the M-PAC framework [31]; however, that work assessed identity as a unidimensional construct despite follow-up work suggesting that it may consist of two factors: exercise beliefs and role identity [42]. Exercise beliefs refer to an individual’s personal convictions about the importance and benefits of exercise in their life. Role identity, on the other hand, is the extent to which a person identifies with being someone who regularly engages in physical activity, considering it a part of their self-concept. Studies examining the two-factor model of exercise identity have shown that role identity is a stronger predictor of exercise cognitions than exercise beliefs [43] and behaviors [42]. In light of these findings, some studies have only used the role identity subscale for brevity purposes [25,26,27]. This approach has resulted in a knowledge gap regarding the understanding of the unique variance in physical activity contributed by each domain of identity within the M-PAC framework. Further investigation is warranted and could provide insight regarding which behavior change techniques may be efficacious for promoting identity development as a mechanism to increase physical activity.

The purpose of the present study was to replicate and extend previous work that examined the predictive utility of the M-PAC framework for explaining self-reported MVPA behavior among adolescents [31]. We sought to extend upon Kwan et al.’s work in two ways: (1) investigating device-measured physical activity behavior and (2) decomposing physical activity identity into two domains to examine the unique contributions of exercise beliefs and role identity for explaining the variance in self-reported and device-measured physical activity behavior. We hypothesized that each level of the M-PAC framework (i.e., reflective, regulatory, reflexive processes) would explain additional variance in physical activity behavior beyond demographic covariates. It was also expected that the predictive utility of the M-PAC framework would be weaker for device-assessed physical activity metrics than self-reported physical activity given that accelerometry captures incidental activities of daily living that may not be predicted by psychological constructs [44]. Finally, based on previous research [42], we predicted that role identity would be a stronger correlate of physical activity behavior than exercise beliefs.

## 2. Methods

### 2.1. Study Design

This study employed a cross-sectional observational design.

### 2.2. Study Setting

Participants were recruited from the three high schools within a suburban school district that served over 10,000 students in a Hispanic-majority metropolitan city in Texas, the United States. Data collection occurred between February 2023 and May 2023.

### 2.3. Participants

A convenience sample of 1849 high school students participated in the present study. Participants were eligible for the present study if they were between the ages of 13 and 18 years, enrolled in one of the three high schools that were sampled, and provided informed consent. Prospective participants were excluded from the present study if they were not fluent in English, experienced a cognitive impairment that would hinder their ability to complete the survey, or had a mobility impairment that would influence accelerometry data collection. Within the overall sample, a subsample of 435 participants agreed to wear an accelerometer for 7 full days.

All participants were entered in a draw for a chance to win one of ten 50 USD gift cards, and those who agreed to participate in the accelerometry component of this study were entered in an additional draw for a chance to win one of ten 50 USD gift cards. All participants completed an online survey on Qualtrics. Responses were screened for double entries; in the case duplicates were identified, the response with more missingness was removed. The demographic component of the survey was presented first, after which all instruments were presented in random order, and participants were unable to backtrack. Those in the accelerometry subsample were fitted with an accelerometer after showing they completed the survey. All participants provided informed consent prior to participation. The study protocol was approved by the school district board as well as an institutional review board (Study #: FY22-23-48).

### 2.4. Variables

Self-reported physical activity. MVPA was assessed using the International Physical Activity Questionnaire-Short Form (IPAQ-SF) [45,46]. Participants responded to four items that assessed the frequency (days) and duration (hours and/or minutes on an average day) of their moderate and vigorous physical activity performed in bouts of at least 10 min over the past seven days. Daily MVPA was calculated by multiplying frequency by duration for moderate and vigorous physical activity, separately, and then summing these products and dividing by seven. As per the scoring rules for the IPAQ-SF, daily MVPA times were capped to 180 min for any participants who exceeded 3 h or 180 min of MVPA per day. The IPAQ-SF has shown acceptable measurement properties when administered among adolescents [47], with prior work validating the instrument against pedometers and accelerometers [48].

Device-assessed physical activity. Device-assessed physical activity was measured using ActiGraph GT3X + triaxial accelerometers (ActiGraph Corp., Pensacola, FL, USA). The wear time period consisted of nine days during which participants were asked to wear the accelerometer on their nondominant wrist for seven full consecutive days (second to eighth day) and to only take the device off for prolonged water immersion. The first (device pick-up) and last day (device return) of the wear period were only partial wear days and were therefore removed from our analyses. The wear time period was carried out during weeks with a regular school schedule. The accelerometers were initialized to sample at 30 Hz, and the subsequent data were downloaded using ActiLife (Version 6.13.5) in GT3X+ file format. Raw accelerometry data files were processed in R and R Studio using the free open-source GGIR package (Version 2.9.0) [49]. Signal processing using the GGIR package was performed according to the default GGIR settings for autocalibration using local gravity as a reference [50], detection of implausible values, and identification of nonwear time. Periods of nonwear time are imputed by default in GGIR, whereby missing data are imputed by the average at similar time points on other days of the week for that participant [51].

Three accelerometry-derived physical activity metrics were computed: average daily MVPA minutes, average daily raw acceleration (physical activity volume), and average daily raw acceleration in the most active hour (physical activity peak-60). Average daily MVPA minutes was computed using Hildebrand et al.’s [52] cut points for segmenting levels of intensity among children/adolescents wearing an ActiGraph device on their nondominant wrist above moderate intensity (≥201.4 milligravitational [mg] units). Raw acceleration-based metrics were computed by converting raw acceleration data (m/s^2^) to one summary measure of acceleration referred to as “Euclidean Norm Minus-One” (ENMO), which is expressed in mg units. ENMO values were reduced to 5 s epochs. Physical activity volume represented the average mg units within a day averaged across all valid days, whereas peak-60 represented the sum of the highest 720 (1 h = 720 5-s epochs) mg unit values recorded within a day, averaged across all valid days. As per previous research, two inclusion criteria were considered valid accelerometry data in the present study: (1) a valid day was defined as at least 10 h of wake-time accelerometer wear [53]; (2) a valid sample was defined as having ≥1 valid day, as this has been determined to be sufficient for generating stable group-level estimates of physical activity in population-level research [54].

Reflective processes: Attitudes. Attitudes were measured using six items and presented on a 7-point bipolar scale as suggested by Ajzen [55] and implemented by Rhodes and Courneya [56]. Each item was prefaced with the stem “Over the next 4 weeks, being physically active, for me, will be…”. Affective attitude was assessed using three items, with responses ranging from unpleasant to pleasant, boring to fun, and unenjoyable to enjoyable. Instrumental attitude was assessed using three items, with responses ranging from harmful to beneficial, bad to good, and useless to useful. Both measures were summed and averaged. Internal consistencies for both instrumental and affective attitude were good (α = 0.94 and 0.94, respectively).

Reflective processes: Perceived behavioral control. Perceived behavioral control was measured using six items that were rated on a 7-point Likert scale, with responses ranging from 1 (strongly disagree) to 7 (strongly agree). This measure included subscales of perceived capability and perceived opportunity as described by Rhodes [57]. Perceived capability consisted of three items such as “I possess the skills to do regular physical activity over the next 4 weeks if I wanted to”. Perceived opportunity also consisted of three items such as “I could find a way to fit it in my schedule so that I do regular physical activity over the next 4 weeks if I wanted to”. Internal consistencies for perceived capability (α = 0.93) and opportunity (α = 0.80) were acceptable.

Regulatory processes: Behavioral regulation. Behavioral regulation was measured using an extended version of the Behavioral Regulation for Physical Activity Scale [58] as per the recommendations of Rhodes [59], which added two additional items for a total of six items to capture a broader range of behavioral regulation tactics. Predictive validity for this instrument was established previously with exercise and physical activity behavior [31,58]. Participants responded to each item on a 7-point Likert scale with responses ranging from 1 (strongly disagree) to 7 (strongly agree). This measure included items that assessed multiple behavioral regulation tactics including self-monitoring, goal setting, action planning, and coping planning. Example items included “I kept track of my physical activity in a diary or log over the last month”, “I set short-term (daily or weekly) goals for leisure-time physical activity last month”, and “I made regular plans concerning when, where, how and what kind of physical activity I did”. Internal consistency was acceptable (α = 0.92).

Reflexive processes: Habit. Habit was measured using the four-item Self-Report Behavioral Automaticity Index (SRBAI) [60]. Convergent and predictive validity for the SRBAI were demonstrated through multiple methods, and content validity was determined by a panel of researchers with expertise in assessing automaticity [60]. Participants responded to each item on a 7-point Likert scale with responses ranging from 1 (strongly disagree) to 7 (strongly agree). Participants were presented with the following statement, “Regular physical activity is something…”, and then responded to the following items: “I do automatically”, “I do without having to consciously remember”, “I do without thinking”, and “I start doing it before I realize I am doing it”. Internal consistency was acceptable (α = 0.92).

Reflexive processes: Identity. Physical activity identity was measured using a modified version of Anderson and Cychosz’s [61] Exercise Identity Scale, which was specific to physical activity. This scale consists of nine items that are rated on a 7-point Likert scale, ranging from 1 (strongly disagree) to 7 (strongly agree). Modification of the Exercise Identity Scale to assess physical activity identity is supported by recent work demonstrating that exercise identity and physical activity identity largely represent a single identity domain [62]. The modified scale included items such as, “I consider myself a physically active person”, “Physical activity is central to my self-concept”, and “Being physically active is something I think about often”. Although exercise identity was originally understood to be a unidimensional construct [61], work since its development suggested that a two-factor model consisting of role identity and exercise beliefs provides a more accurate representation of this construct [42]. We therefore decided to decompose the unidimensional scale to examine the three-item role identity and six-item exercise beliefs subscales as well. Internal consistency for the full scale (α = 0.95) as well as the role identity (α = 0.91) and exercise beliefs (α = 0.93) subscales was acceptable. Structural and criterion validity (against physical activity behavior) of the Exercise Identity Scale have been established previously [42].

Covariates. Self-reported biological sex (male/female), race/ethnicity (Hispanic/Black/White/Other), age, and body mass index classification based on CDC growth curves (underweight/normal weight/overweight/obese; [63]) were included in our models as covariates. Each of these variables has been found to be correlated with physical activity during adolescence [64].

### 2.5. Bias

To reduce social desirability bias, participants were informed that their responses to any of the questions and how much physical activity they engaged in if they completed the accelerometry component of this study would not influence their odds of winning a gift card.

### 2.6. Study Size

As the broader goal of this collaborative project was to assess physical activity behavior among as many students within the school district as possible, a sample size calculation was not performed, and any students who expressed interest in the study were invited to participate pending informed consent.

## 3. Data Analysis

All analyses were performed in R (version 4.3.0) and R Studio (version 2023.06.0, PBC, Boston, MA, USA) using the mice [65], miceadds [66], robustbase [67], and ltm [68] packages. First, summary scores were computed for the IPAQ-SF as per its scoring rules and each M-PAC process measure, which were summed and averaged across the number of items for each respective measure. Next, we inspected the data for missingness using the mice and miceadds packages. Data were considered missing at random, and multiple imputation by chained equations was computed using the mice and miceadds packages to replace missing values. Multiple imputation is considered a best practice for handling missing data [69]. As per recommendations to set m > 100 times the highest fraction of missing information (self-reported MVPA dataset: 18% for perceived opportunity; device-assessed physical activity dataset: 24% for physical activity) [70], a total of 18 multiply imputed datasets were created for the self-reported MVPA dataset, and 25 datasets were created for the accelerometry subsample.

First, correlation matrices were computed for self-reported and device-measured physical activity with each of the M-PAC framework constructs. Prior to conducting our analyses, all relevant assumptions were tested. Assumptions for linearity, normality, and homogeneity of variance were met; however, multivariate outliers were observed in each model. Since ordinary least squares regression estimates are sensitive to outliers and highly influential observations, a robust estimator was employed to reduce the influence of these observations. This approach decreases the weights of observations with large residuals to reduce their influence on model estimates. Furthermore, although students were nested within three schools, for confidentiality purposes, school-level data were not recorded; therefore, multilevel models were not able to be computed to handle the violation of independence. Next, a series of hierarchical robust linear regression models were computed to predict self-reported MVPA and the three device-assessed physical activity metrics. Model 1 included only the covariates as predictors. Model 2 also included the reflective process measures of affective and instrumental attitudes as well as perceived capability and opportunity. Model 3 also included the regulatory process measure of behavioral regulation. In Model 4, we added the reflexive process measures of habit and physical activity identity. In Model 5, we replaced the unidimensional physical activity identity scale with its two subscales: role identity and physical activity beliefs. Beta coefficients and standard errors were computed for each variable. Adjusted *R*^2^ and *R*^2^ change values were computed for each model to determine the proportion of variance in the dependent variable that was uniquely attributed to each successive layer of processes in the M-PAC framework. Statistical significance was set at *p* < 0.05.

## 4. Results

The overall sample was, on average, 16.0 ± 1.22 years of age, 52.3% girls, with participants identifying as 87.8% Hispanic, 4.3% Black, 4.2% White, and 3.6% Other. Based on self-reported height and weight, 53.8% of participants were classified as normal weight, 2.7% as underweight, 19.7% as overweight, and 23.7% as obese as per CDC curve percentiles [63]. There was a similar demographic profile in the accelerometry subsample (mean age = 15.90 ± 1.22 years; 52.3% girls; 88.2% Hispanic, 4.8% White, 3.8% Black, 3.2% Other): 49.8% of participants were classified as normal weight, 2.9% as underweight, 21.8% as overweight, and 25.4% as obese. Missing data ranged from 0.2% for age and biological sex to 25.8% for perceived capability in the self-reported dataset, whereas missingness ranged from 2.5% for age and biological sex to 25.7% for device-measured physical activity among the accelerometry subsample. Of the 323 participants who had a valid accelerometry sample (*n* = 112 did not meet the inclusion criteria and thus had their data imputed), an average of 4.02 total valid days were recorded, with an average awake wear time of 879 ± 124 SD minutes and an average nonwear time percentage of 10.6% ± 13.3 SD.

For the full sample, adolescents self-reported engaging in, on average, 68.67 ± 62.10 SD minutes of daily MVPA (i.e., 480.74 ± 434.70 SD minutes/week). In contrast, however, accelerometry estimates suggested that the sample was much less active, averaging 15.10 ± 24.24 SD minutes of daily MVPA. It should be noted, however, that the correlation between self-reported and device-assessed MVPA was small (*r* = 0.15) among the participants that completed both measures. Among the full sample, a closer inspection of the M-PAC framework processes revealed participants endorsed somewhat strong instrumental (M = 5.29 on a seven-point scale) and affective (M = 4.88) attitudes as well as perceived capability (M = 4.77). Perceived opportunity (M = 4.42 on a seven-point scale), behavioral regulation (M = 3.59), habit (M = 3.99), and identity (M = 3.9) were endorsed as relatively moderate, with average scores oriented around the neutral anchor on the Likert scale. The M-PAC framework variable values were slightly higher among the accelerometry subsample. Complete details of the descriptive statistics, including correlation matrices, are presented in Table 1 and Table 2.

### 4.1. Self-Reported Average Daily MVPA

**Figure 1:**
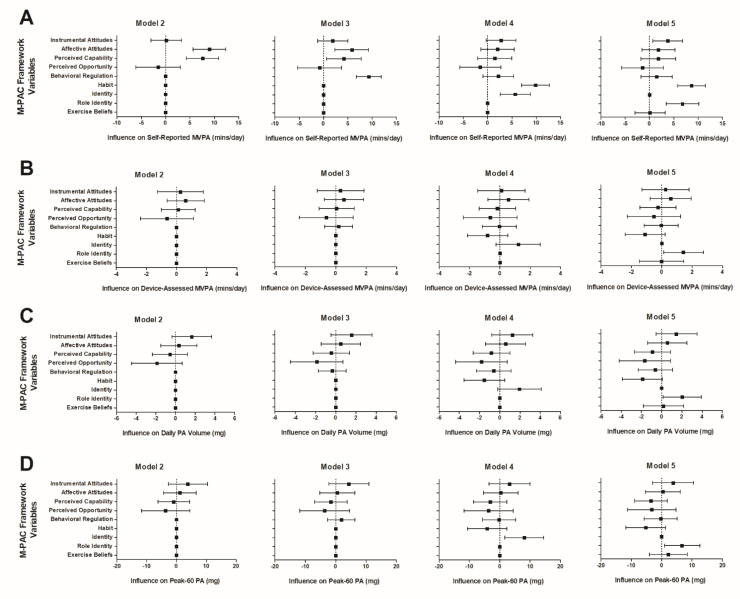
Beta coefficients and 95% CIs for associations between Multi-Process Action Control framework constructs and average daily self-reported MVPA (**A**), device-assessed MVPA (**B**), physical activity volume (**C**), and peak-60 physical activity (**D**). Model 2 = reflective processes; Model 3 = reflective and regulatory processes; Model 4 = reflective, regulatory, and reflexive processes; Model 5 = reflective, regulatory, and reflexive processes with identity decomposed into role identity and exercise beliefs, adjusted for covariates.

The results for Model 1 (covariates only) explained 6.5% of the variance in the self-reported MVPA. Adding reflective processes (Model 2) explained an additional 12% of the variance in the self-reported MVPA, which was a significant increase from that in Model 1 (*p* < 0.001), with affective attitudes (B = 8.99 ± 1.72 SE, *p* < 0.001) and perceived capability (B = 7.59 ± 1.69 SE, *p* < 0.001) being significant predictors. Model 3 integrated regulatory processes, which explained an additional 3.5% of the variance in the self-reported MVPA, which was a significant increase from that in Model 2 (*p* < 0.001). Affective attitudes (B = 5.81 ± 1.77 SE, *p* = 0.001), perceived capability (B = 4.19 ± 1.82 SE, *p* = 0.025), and behavioral regulation (B = 9.28 ± 1.32 SE, *p* < 0.001) were significant predictors of self-reported MVPA in Model 3. Finally, adding reflexive processes significantly increased the variance explained in the self-reported MVPA by 4.8%, which was a significant increase from that in Model 3 (*p* < 0.001), and indicated that habit (B = 9.85 ± 1.46 SE, *p* < 0.001) and identity (B = 5.69 ± 1.58 SE, *p* < 0.001) were significant predictors. Decomposing identity into role identity and physical activity beliefs (Model 5) explained an additional 6% of the variance in the self-reported MVPA beyond that in Model 3 (*p* < 0.001) and indicated that instrumental attitudes (B = 3.77 ± 1.58 SE, *p* = 0.018), habit (B = 8.66 ± 1.46 SE, *p* < 0.001), and role identity (B = 6.78 ± 1.70 SE, *p* < 0.001) were significant predictors. In total, the full models explained 26.8% (Model 4) and 28.0% (Model 5) of the variance in the self-reported daily MVPA, which included 20.3% (Model 4) and 21.5% (Model 5) of the variance beyond the covariates. The results are presented visually in Figure 1A.

### 4.2. Device-Assessed Physical Activity

Average daily MVPA. The results for Model 1 (covariates only) explained 2.7% of the variance in the average daily MVPA. Model 2 explained an additional 2.4% of the variance in the average daily MVPA, but this was a nonsignificant increase (*p* = 0.35) from that in Model 1. None of the reflective processes were significant predictors. In adding regulatory processes, Model 3 explained an additional 0.1% of the variance in the average daily MVPA, which was a nonsignificant increase in the variance explained (*p* = 0.55). None of the M-PAC variables were significant predictors in Model 3. Model 4 integrated the reflexive processes, which explained an additional 1.7% of the variance in the average daily MVPA, which was a significant increase in the variance explained (*p* < 0.001), although none of the M-PAC variables were significant predictors. Finally, decomposing identity into its two subscales (Model 5) explained an additional 3.2% of the variance in the average daily MVPA beyond that in Model 3, which was a significant increase (*p* < 0.001), and indicated that role identity was a significant predictor (B = 1.43 ± 0.67 SE, *p* = 0.036). In total, the full models explained 6.9% (Model 4) and 8.4% (Model 5) of the variance in the average daily MVPA time, which included 4.2% (Model 4) and 5.7% (Model 5) of the variance beyond the covariates. The results are presented visually in Figure 1B.

Physical activity volume. The results for Model 1 (covariates only) explained 4.0% of the variance in the physical activity volume. Model 2 explained an additional 3.3% of the variance in the physical activity volume, which was a significant increase (*p* = 0.02), although none of the reflective processes were significant predictors. In adding regulatory processes, Model 3 explained an additional 0.1% of the variance in the physical activity volume, which was a nonsignificant increase (*p* = 0.68). None of the M-PAC variables were significant predictors in Model 3. Model 4 integrated the reflexive processes, which explained an additional 2.8% of the variance in the physical activity volume, which was a significant increase (*p* < 0.001), although none of the M-PAC variables were significant predictors. Finally, decomposing identity into its two subscales (Model 5) explained an additional 4.4% of the variance in the physical activity volume beyond that in Model 3, which was a significant increase (*p* < 0.001), and indicated that role identity was a significant predictor (B = 2.03 ± 0.95 SE, *p* = 0.038). In total, the full models explained 10.2% (Model 4) and 11.8% (Model 5) of the variance in the physical activity volume, which included 6.2% (Model 4) and 7.8% (Model 5) of the variance beyond the covariates. The results are presented visually in Figure 1C.

Peak-60 physical activity. The results for Model 1 (covariates only) explained 3.4% of the variance in the peak-60 physical activity. Model 2 explained an additional 1.9% of the variance in the peak-60 physical activity, which was a nonsignificant increase (*p* = 0.13). None of the reflective processes were significant predictors in Model 2. In adding regulatory processes, Model 3 explained an additional 0.3% of the variance in the peak-60 physical activity, which was a nonsignificant increase (*p* = 0.11). None of the M-PAC variables were significant predictors in Model 3. Model 4 integrated the reflexive processes, which explained a significant increase (2.9%, *p* < 0.001) in the variance in the peak-60 physical activity, and indicated that identity (B = 8.04 ± 3.27 SE, *p* = 0.017) was a significant predictor. Finally, decomposing identity into its two subscales (Model 5) explained an additional 4.1% of the variance in the peak-60 physical activity beyond that in Model 3, which was a significant increase (*p* < 0.001), and indicated that role identity was a significant predictor (B = 6.73 ± 2.96 SE, *p* = 0.026). In total, the full models explained 8.5% (Model 4) and 9.7% (Model 5) of the variance in the peak-60 physical activity, which included 5.1% (Model 4) and 6.3% (Model 5) of the variance beyond the covariates. The results are presented visually in Figure 1D.

## 5. Discussion

The present study aimed to replicate and extend upon Kwan et al.’s [31] work investigating the predictive utility of the M-PAC framework for physical activity behavior in adolescents. The findings for both self-reported MVPA and device-assessed physical activity metrics aligned with those of previous research in that that each successive layer in the M-PAC framework explained additional variance in physical activity behavior, although the M-PAC framework only had roughly a third of the predictive power for device-assessed behavior (*R*^2^ = 6.9–11.8%) compared to self-reported behavior (*R*^2^ = 26.8–28.0%). A closer inspection of the variables within the M-PAC framework revealed that reflexive processes (i.e., habit, identity) were the most robust predictors of physical activity behavior and extend the current knowledge by showing that role identity appears to be a particularly salient correlate of physical activity behavior in adolescents. A key feature of understanding the correlates of physical activity behavior is informing the development of interventions. These findings suggest that targeting individuals with low role identity may hold promise, which is particularly relevant during adolescence, given it is a life stage during which self-definitions are especially malleable [32].

Very few studies have examined the predictive utility of models that integrate reflexive processes into a comprehensive framework to understand physical activity behavior, let alone that among adolescents. Albeit to a weaker extent based on the variance explained (20.3% versus 26.1% beyond covariates), our findings replicate those of Kwan et al. [31] in that each successive layer of the M-PAC framework explained additional variance in self-reported MVPA among adolescents. Furthermore, reflexive processes (i.e., habit and identity) were the strongest predictors. Similar additive increases in variance explained were observed across studies in that adding reflective processes to the model resulted in the largest increase, followed by reflexive processes, and lastly, regulatory processes [31]. One potential explanation for the disparity in the variance explained between the studies may be the racial/ethnic differences of the samples. Whereas the original study involved a majority White (52.4%) sample of Canadian adolescents and very few Hispanics (4.3%), the present sample consisted of American adolescents who primarily identified as Hispanic (87.8% versus 4.2% White). From this perspective, the same cultural influences, attitudes, and beliefs that have been cited to explain why rates of obesity are highest among Hispanic youth may have played a role in these observed differences [71]. This notion is supported by evidence of lower average scores on each of the M-PAC variables in our study compared to those observed by Kwan et al. [31]. Future work with a more diverse sample is warranted for examining potential ethno-racial differences. It is also interesting to note that instrumental attitudes were a significant correlate of the self-reported MVPA in the final model (Model 5) with the identity scale decomposed. Previous studies have generally shown that most adolescents are aware of the health benefits of physical activity (as evidenced by strong scores on measures of instrumental attitudes), despite different levels of behavioral uptake [6,72,73], often rendering this association nonsignificant [21]. It is therefore plausible that there is greater concordance between Hispanic adolescents’ attitudes regarding the expected utility of physical activity and their actual behavior, which may be explained by cultural factors. For example, in Hispanic culture, adolescents have many family obligations, which has been shown to detrimentally affect MVPA levels [74]. Taken together, given that Hispanic adolescents meet the public health recommendations for physical activity at lower rates than their White counterparts [75,76], culturally tailored interventions that target specific variables within the M-PAC framework may be well suited for promoting physical activity adoption and maintenance based on where individuals are in the action control process (i.e., behavioral initiation to continuation).

Extending Kwan et al.’s [31] work by investigating the predictive utility of the M-PAC framework for device-assessed physical activity metrics revealed a stark difference in how much variance may be explained. Specifically, the best predictive model for the device-based metrics only explained an additional 7.8% of the variance in the physical activity volume (i.e., overall raw acceleration) beyond the initial covariate-only model, which was roughly one-third of what was observed for the best model when the self-reported MVPA was the outcome (21.5% beyond covariates). These findings highlight the challenges in explaining physical activity behavior using only psychological variables. One explanation for the discrepancy in the variance explained across the physical activity measures is that accelerometry captures actual movement in contrast to self-reports, which can be prone to individuals relying on heuristics related to the duration of the activity when providing estimates of their behavior. For example, research using accelerometry has shown that students are only active for a small proportion of the time during physical education classes [77], but self-reported estimates might consider the entire physical education period (e.g., 50 min) as minutes engaged in MVPA. This discordance between device-assessed and self-reported physical activity is well established [78]. Second, incidental activity that simply occurs as a part of daily life (e.g., household chores, walking between classes) beyond volitional behaviors is also captured via accelerometry. Such movements may not be as well correlated with the variables outlined in theoretical frameworks, as these behaviors are not captured in the original operationalization of physical activity and therefore reduce the precision in the associations between psychological correlates and device-assessed physical activity metrics. Emerging machine learning algorithms for processing accelerometry data may be useful for identifying purposeful bouts of specific activities, potentially enhancing the precision of our estimates [79]. Signal–contingent ecological momentary assessments paired with accelerometry offers another promising methodology to gather contextual information about physical (in)activity and its relationship to psychological correlates [80]. Regardless, the current findings suggest that the predictive utility of the M-PAC framework is weaker for device-assessed physical activity behavior than that of self-reported instruments.

Beyond the overall variance in self-reported and device-assessed physical activity behavior, it is important to acknowledge which variables within the M-PAC framework were the strongest predictors of behavior. Our findings aligned with those of previous work in that the reflexive processes (habit and identity) were the strongest predictors of the self-reported MVPA among adolescents [31], addressing a knowledge gap by showing that role identity—but not physical activity beliefs—is driving the significant association for identity. The models examining device-assessed physical activity metrics partially aligned with the self-reported results in that role identity was the only significant predictor. Collectively, these findings support those in the literature indicating that role identity is a stronger correlate of physical activity behavior than physical activity beliefs [42]. Observing role identity as a consistent predictor of device-assessed physical activity behavior is an important finding in light of previous work suggesting that individuals with a strong physical activity identity overestimate their physical activity behavior to correspond with the importance of their identity [39]. Looking closer at the bivariate correlations, however, only revealed a significant association between role identity and peak-60 physical activity, and the effect was small (*r* = 0.12), which was roughly one-third of the size of the medium correlation observed for the self-reported MVPA in the present study (*r* = 0.37) and meta-analytic evidence for device-assessed physical activity from four studies on adults (*r =* 0.32) [81]. Adolescence, a formative period in which identities are shaped [32], highlights the potential for behavior change interventions targeting role identity to promote sustained physically active lifestyles during this life stage. Recent work has suggested that behavior change techniques such as identity associated with changed behavior (e.g., assessments of one’s identity surrounding being physically active, including clothing choices and social media posts) might be ideal to target as they could tap into one’s role identity [82]. This idea is further supported by a recent prospective study that showed that physical activity identity is a significant predictor of future adolescent MVPA assessed one year later [83]. The preliminary findings of interventions provide support for targeting physical activity identity to increase physical activity [20,84]. However, more work is needed to integrate cultural values into behavior change techniques, as cultural stress and acculturation have been shown to impact identity formation in Hispanic adolescents [74]. Although the findings for habit were only supported for self-reported physical activity, behavior change techniques such as providing (e.g., visual reminders that every move counts) and altering (e.g., redesigning study spaces to have walking desks) cues may be a promising approach for promoting physical activity behavior among this population [85]. Considering that theories of physical activity behavior can provide a roadmap for intervention development, the present findings emphasize the importance of reflexive processes in intervention frameworks for mitigating declines in physical activity during adolescent development.

While the present study is the first to replicate and build on the existing literature applying the M-PAC framework, it is not without limitations. First, we employed a cross-sectional design, and thus the associations do not provide any information pertaining to directionality. While at least one prospective study has used the M-PAC framework to predict changes in adolescents’ physical activity behavior over time [30], future studies that employ longitudinal designs with advanced analytical techniques are needed to model the interrelations between constructs within the M-PAC framework and how they relate to physical activity [86]. A further limitation of the cross-sectional design relates to the incongruence between the period of reference for the reflective M-PAC framework constructs (i.e., “Over the next 4 weeks…”) and physical activity behavior (previous seven days for the self-reported instrument versus the next seven days for accelerometry) [87]. As the present study sought to replicate and extend upon previous findings, the frame of reference for the M-PAC framework constructs was selected for consistency with the methods employed by Kwan et al. [31]. Despite this concern, the stability of the reflective processes observed by Kovacevic et al. [30] over a one-year period among adolescents suggests that the compatibility of the reference period may have had limited effects on the associations between attitudes and perceived behavior control with physical activity behavior in the present study. Third, convenience sampling was employed, which risks bias toward including individuals interested in participating in a study focused on physical activity. Fourth, there are also important limitations to acknowledge regarding physical activity measurement. Despite their ease of implementation with large samples, self-reported physical activity instruments are prone to bias and recall errors [37]. Using accelerometry can improve the precision of physical activity estimates [38], but accelerometry is unable to capture all types of activities (e.g., cycling, resistance training), can be prone to issues with protocol compliance (i.e., nonwear time), and data collection may be influenced by participant interference (e.g., behavior changes due to monitoring, device shaking) [41]. Fifth, there is limited evidence regarding the validity of the instruments used to assess attitudes and perceived behavioral control, although these instruments were developed based on earlier recommendations by Ajzen [55] and have been used extensively in the field of exercise psychology when conducting studies couched within the M-PAC framework based on recommendations by Rhodes [59]. Finally, for confidentiality purposes, information about the school in which students were enrolled and socioeconomic status indicators were not assessed. Accounting for the nested structure of the data (i.e., students nested within three schools) would have been the most appropriate from a modeling standpoint and could have resulted in different findings. Socioeconomic status indicators such as household income or parental education are also well known to be correlated with adolescent physical activity [64], and their inclusion could have also adjusted the results. Unfortunately, after discussions with key stakeholders in the school district, we were unable to ascertain some of this critical information.

In conclusion, the present study replicated and extended Kwan et al.’s [31] work examining the predictive utility of the M-PAC framework in explaining adolescents’ physical activity behavior. While the present study generally observed similar, albeit slightly weaker, results for self-reported MVPA in comparison to that of Kwan et al. [31], it is noteworthy that the M-PAC framework explained considerably less variance in device-assessed physical activity metrics versus than the self-reported instruments. These findings underscore the difficulty of explaining adolescents’ physical activity using only variables at the intrapersonal level when multiple levels of influence (i.e., interpersonal, environmental) are known to play a role. Within the M-PAC framework, role identity was found to be the most consistent predictor of physical activity behavior, which suggests that behavior change techniques that facilitate physical activity identity formation and prevent foreclosure may be a key ingredient in physical activity interventions targeting adolescents. Moving forward, longitudinal studies are required to understand the dynamic nature of how these psychological processes evolve and influence one another as well as physical activity over time so that interventions can be tailored to the individual to provide the right type and amount of support when it is needed.

## Figures and Tables

**Table 1:** Correlation matrix of Multi-Process Action Control Framework variables and self-reported daily moderate-to-vigorous physical activity.

	1.	2.	3.	4.	5.	6.	7.	8.	9.	10.	M (SD)
1. Instrumental Attitudes	-										5.29 (1.53)
2. Affective Attitudes	**0.78**	-									4.88 (1.58)
3. Perceived Capability	**0.54**	**0.54**	-								4.77 (1.54)
4. Perceived Opportunity	**0.44**	**0.38**	**0.60**	-							4.42 (0.91)
5. Behavioral Regulation	**0.27**	**0.39**	**0.45**	**0.24**	-						3.59 (1.49)
6. Habit	**0.33**	**0.46**	**0.48**	**0.30**	**0.48**	-					3.99 (1.53)
7. Identity	**0.44**	**0.53**	**0.54**	**0.35**	**0.65**	**0.61**	-				3.90 (1.51)
8. Role Identity	**0.26**	**0.40**	**0.41**	**0.24**	**0.61**	**0.60**	**0.82**	-			3.58 (1.73)
9. PA Beliefs	**0.47**	**0.53**	**0.53**	**0.35**	**0.57**	**0.52**	**0.95**	**0.60**	-		4.06 (1.64)
10. Daily MVPA	**0.26**	**0.31**	**0.31**	**0.18**	**0.33**	**0.40**	**0.37**	**0.37**	**0.32**	-	68.67 (62.10)

Note: Bolded values = *p* < 0.05.

**Table 2:** Correlation matrix of Multi-Process Action Control Framework variables and device-assessed physical activity metrics.

	1.	2.	3.	4.	5.	6.	7.	8.	9.	10.	11.	12.	M (SD)
1. Instrumental Attitudes	-												5.40 (1.49)
2. Affective Attitudes	**0.77**	-											5.03 (1.56)
3. Perceived Capability	**0.48**	**0.52**	-										5.03 (1.38)
4. Perceived Opportunity	**0.42**	**0.54**	**0.54**	-									4.56 (0.84)
5. Behavioral Regulation	**0.23**	**0.40**	**0.42**	**0.21**	-								3.75 (1.48)
6. Habit	**0.27**	**0.44**	**0.41**	**0.25**	**0.59**	-							4.15 (1.42)
7. Identity	**0.44**	**0.54**	**0.54**	**0.32**	**0.63**	**0.58**	-						4.06 (1.53)
8. Role Identity	**0.31**	**0.44**	**0.47**	**0.24**	**0.59**	**0.59**	**0.85**	-					3.74 (1.74)
9. PA Beliefs	**0.46**	**0.53**	**0.52**	**0.32**	**0.58**	**0.51**	**0.96**	**0.66**	-				4.22 (1.63)
10. Daily MVPA	0.01	0.04	0.07	−0.05	0.09	0.08	0.07	0.09	0.05	-			15.10 (24.24)
11. PA Volume	0.09	0.09	0.00	−0.08	0.03	0.01	0.07	0.09	0.05	**0.83**	-		35.14 (17.55)
12. Peak-60 PA	0.06	0.09	0.07	0.00	0.11	0.03	0.10	**0.12**	0.08	**0.79**	**0.78**	-	110.22 (78.40)

Note: Bolded values = *p* < 0.05.

## Data Availability

Data are available upon reasonable request from Denver Brown (denverbrown@ksu.edu).
